# Applications of Forced Oscillatory Technique in Obstructive and Restrictive Pulmonary Diseases: A Concise State of the Art

**DOI:** 10.3390/jcm14165718

**Published:** 2025-08-12

**Authors:** Claudio Tirelli, Sabrina Mira, Marta Italia, Sara Maggioni, Carmelo Intravaia, Martina Zava, Simone Contino, Elena Maria Parazzini, Michele Mondoni

**Affiliations:** Respiratory Unit, ASST Santi Paolo e Carlo, Department of Health Sciences, Università degli Studi di Milano, 20142 Milan, Italy

**Keywords:** Forced Oscillatory Technique (FOT), obstructive lung disease, restrictive lung disease, asthma, COPD, ILD, obesity, kyphoscoliosis, neuromuscular disorders

## Abstract

The FOT is a non-invasive method for assessing respiratory mechanics. It enables the measurement of respiratory system impedance by applying pressure oscillations through a loudspeaker at the subject mouth and then studying its deformation, which is commensurate to the resistance opposed by the respiratory system. The main parameters which can be determined with the FOT are the impedance (Z) and the components of respiratory resistance (Rrs) and reactance (Xrs). The FOT has been predominantly applied to the study of respiratory mechanics for research purposes; however, preclinical experiments and subsequently observational clinical studies have demonstrated that FOT can effectively assess airway obstruction, bronchodilator response, bronchial hyperresponsiveness, and the presence of small airways disease. More recently, studies on the FOT in restrictive lung diseases have also been reported. Nonetheless, international guidelines on the precise applications of the FOT in lung diseases are still lacking. The aim of the review was to describe the technical aspects related to the FOT methodology in clinical practice and to provide a concise state of the art on the applications of the FOT in obstructive and restrictive lung diseases.

## 1. Introduction

The Forced Oscillatory Technique (FOT) is a non-invasive diagnostic tool that measures respiratory impedance (Z), and provides information on airway reactance (Xrs) and resistance (Rrs) across different airways’ regions [1].

First described by DuBois et al. in 1956 in the attempt to study the resistive and elastic features of the respiratory system, the FOT has then been applied in research activities, mainly in pre-clinical settings, to characterize the mechanics of the respiratory system [2,3,4]. At the beginning, studies on animals were mainly concentrated on laboratory rodents, then the technique was tried also on larger animals and, finally, on humans [5,6]. The great potential of the FOT to probe the lung mechanics in a very effortless way represented a major advantage which contributed to its wider adoption. Indeed, lung function testing is traditionally based on the use of spirometry, which is an effort-dependent technique. Spirometry can often be challenging to perform by older or very young people. On the contrary, through the application of a defined range of frequencies, the FOT can measure respiratory impedance, resistance, and reactance by superimposing external oscillatory pressure waves over spontaneous breathing [7]. Moreover, central and peripheral airway function can be studied with the FOT frequency-dependent analysis, thus leading to its application both in obstructive and restrictive respiratory syndromes, from asthma and chronic obstructive pulmonary disease (COPD) to interstitial lung disease (ILD) [8,9]. The FOT has proven particularly useful in identifying small airways disease (SAD), especially in severe asthma, where early detection of SAD could help in treatment decisions and, thus, improve outcomes [10,11].

**Figure 1:**
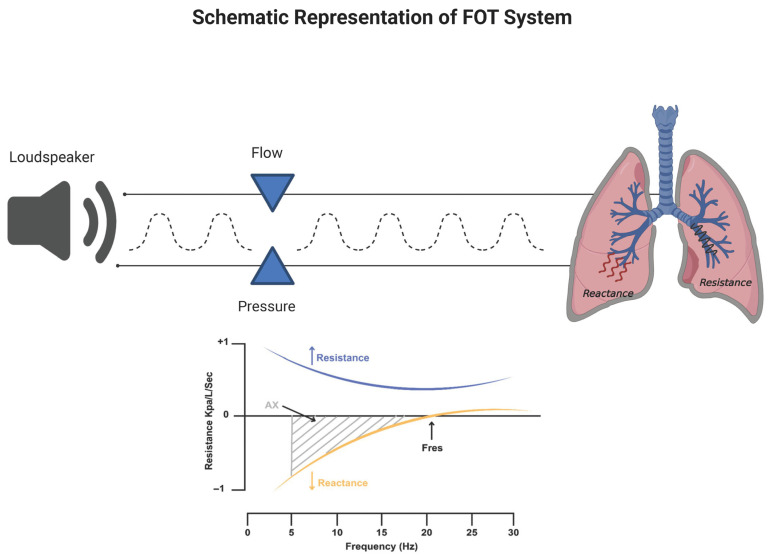
Applications of the FOT in obstructive and restrictive lung diseases.

The aim of the review was to describe the technical aspects related to FOT methodology and provide a concise overview on the main clinical applications of the FOT in obstructive and restrictive lung disease (Figure 1).

## 2. Forced Oscillatory Technique: Methodology and Technical Aspects

**Figure 2:**
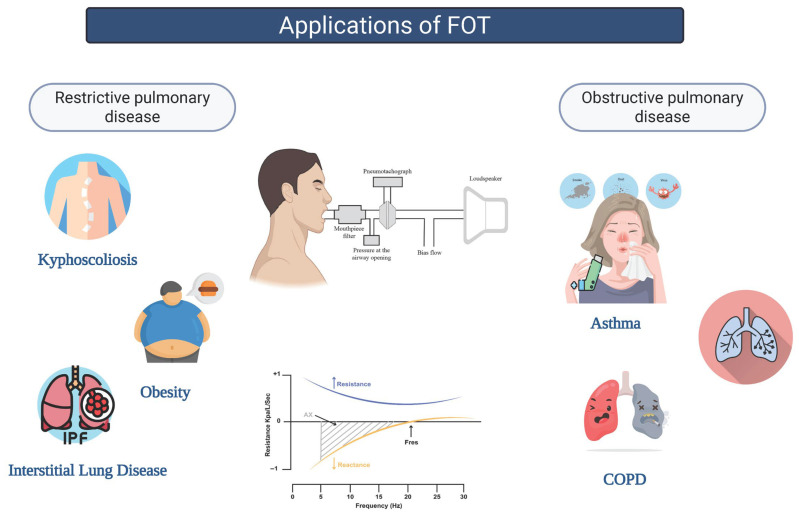
**Schematic representation of the Oscillometry system and Oscillometry impedance plot.** The plot depicts the Impedance of the respiratory system (Z) versus frequency in hertz (Hz); the upper curve represents the Resistance (Rrs) and the lower curve the Reactance (Xrs) of the respiratory system.

The FOT is a non-invasive diagnostic method for assessing respiratory mechanics. It enables the measurement of respiratory system impedance by applying pressure oscillations to the subject during spontaneous breathing with open mouth [12] (Figure 2).

### 2.1. Methodology

More specifically, mechanical information about the respiratory system can be collected by imposing an oscillatory waveform signal through a loudspeaker at the subject’s mouth and then studying its deformation, which is commensurate with the resistance opposed by the respiratory system.

During the procedure, the patient breathes normally through a mouthpiece connected to a system that both generates and detects oscillatory signals, while supporting their cheeks with their hands to reduce upper airway shunt and interference from orofacial structures [13,14].

An external pressure oscillation, with a typical range of frequencies (i.e., 5–35 Hz), is superimposed on spontaneous breathing through a mouthpiece. The oscillations are then recorded by a transducer system, and the resulting pressure-flow relationship is analyzed (i.e., Fourier transformation) to calculate respiratory impedance.

### 2.2. FOT Parameters

The impedance (usually abbreviated by the letter Z) reflects the overall opposition to the oscillation wave determined by the airways. For clinical applications, the output signal Z is recorded at the same physical location where the input signal is delivered, i.e., the mouth [15,16]. When performing the FOT in humans, it is strictly recommended to adopt oscillation frequencies which are outside the frequency of the subject normal breathing in order to avoid contamination in the spectral content of the input signal. In humans, clinical devices are typically set to adopt 5 Hz as the lowest frequency, because it must be at least an order of magnitude above the frequency of spontaneous breathing (0.2–0.3 Hz) [17]. In very limited conditions, such as anesthetized paralyzed children, mechanical ventilation, voluntary or induced apnea, low-frequency measurements can be used [18,19]. The impedance is composed of respiratory resistance (Rrs) and reactance (Xrs) [13]. These two fundamental parameters can be derived by the FOT as well. While Rrs reflects the total opposition to airflow due to airway caliber and tissue friction, Xrs reflects tissue elasticity and inertance of air [20,21]. Measurements are usually taken over a few breaths, lasting approximately 15–30 s. The technique does not require forced expiratory maneuvers, so it is particularly useful in populations such as young children, elderly individuals, and patients with neuromuscular disorders. Rrs represents the energy dissipative component of impedance and primarily reflects airway resistance [22]. Rrs is commonly measured at standard frequencies such as 5 Hz (R5), representing total airway resistance (central and peripheral), and 20 Hz (R20), which reflects central airway resistance [23,24]. The difference between R5 and R20 (R5–20) is used as an index of ventilation heterogeneity or small airway obstruction [7,24]. Xrs, in contrast, represents the elastic and inertial components of impedance. More negative Xrs values at low frequencies, particularly at 5 Hz (X5), are indicative of increased lung stiffness or reduced compliance of the peripheral lung [7,24]. Additionally derived parameters include the resonant frequency (Fres), which is the frequency at which Xrs crosses zero, and the area under the reactance curve (AX), calculated from X5 to Fres. These parameters provide further insight into the severity of mechanical impairment [13].

### 2.3. Recommendations for Measurements

The FOT is characterized by high reproducibility and the advantage of being effort-independent, as it does not require maximal respiratory maneuvers from the patient. This makes it particularly well-suited for children, elderly individuals, and patients with severe respiratory impairment [7,14]. Studies have shown that the FOT is feasible in children as young as two years old and demonstrates higher success rates compared to spirometry in young children with asthma [25]. The technique has been applied effectively to assess airway obstruction, bronchodilator response, and bronchial hyperresponsiveness, especially in pediatric populations with asthma, cystic fibrosis, and chronic lung disease of prematurity [14,24]. Furthermore, its sensitivity in detecting peripheral airway dysfunction may exceed that of traditional spirometry, making it a promising tool for both diagnosis and long-term monitoring [14,25]. Despite these advantages, the clinical implementation of the FOT still faces challenges related to standardization across devices and interpretation of parameters. Scientific societies are developing standardized methods to perform and interpret FOT measurements so as to promote wider use of the FOT in routine clinical practice [24,25].

A schematic summary of the trends in FOT parameters together with FOT clinical and research applications in obstructive and restrictive lung diseases are displayed in Table 1.

## 3. Application of the Forced Oscillatory Technique in Obstructive Lung Diseases

The FOT has been thoroughly studied in the context of obstructive lung diseases, i.e., asthma and chronic obstructive pulmonary disease (COPD). The typical FOT pattern includes increased respiratory system resistance (Rrs) and decreased reactance (Xrs), both of which correlate with the degree of airflow obstruction as assessed by spirometry [26,27].

### 3.1. Asthma

Asthma is a chronic inflammatory disorder of the airways characterized by variable and recurring symptoms, reversible airflow obstruction, and airway hyperresponsiveness [28]. Clinical manifestations typically include episodes of wheezing, coughing, chest tightness, and dyspnea. These symptoms may vary in frequency and severity, often worsening at night or during physical exertion. Acute exacerbations may be triggered by inhaled allergens, pollutants, or irritants, leading to bronchoconstriction, mucus hypersecretion, and impaired oxygen exchange [29]. Diagnosis is primarily based on a characteristic pattern of symptoms and their response to treatment, and spirometry serves as the gold standard for diagnostic confirmation by demonstrating reversible airflow limitation. However, in children under six years of age, spirometry is often unreliable due to poor technique, necessitating alternative diagnostic tools. The FOT has emerged as a valuable adjunct to spirometry. Unlike spirometry, which requires forced expiratory maneuvers, the FOT measures respiratory impedance during tidal breathing, making it especially suitable for pediatric and elderly people where standard spirometry is challenging.

To date, limited studies have demonstrated clear diagnostic superiority of either technique in asthma. Notably, one recent investigation reported that oscillometry outperformed spirometry in both diagnosing asthma and assessing response to treatment over a three-month period [30]. Conversely, another study involving military personnel found that spirometry was more effective than oscillometry in diagnosing obstructive lung disease [31]. Such variability may stem from heterogeneity in device types and the lack of universally accepted abnormal threshold values for oscillometric parameters. The use of oscillometry for bronchodilator and bronchoprovocation testing in asthma is an area of investigation. According to current technical standards, a significant bronchodilator response (BDR) is considererd by a 40% decrease in R5, a 50% increase in X5, and an 80% decrease in the area under the reactance curve (Ax) in both adults and children [20]. A recent study assessing the predictive value of different oscillometric BDR thresholds found that resonant frequency (Fres) demonstrated the strongest correlation with positive BDR by spirometry and was more sensitive in identifying uncontrolled asthma [32]. Due to considerable variability in reported outcomes, the technical standards document does not currently provide definitive cut-off values for bronchoprovocation testing [20]. One of the major advantages of oscillometry lies in its heightened sensitivity for detecting bronchoconstriction [33]. This has been corroborated by recent studies demonstrating that oscillometry requires lower methacholine doses to achieve a positive response, and that baseline abnormal oscillometric measurements predict positive methacholine challenge results, as determined by spirometry [34]. In children undergoing evaluation for exercise-induced bronchoconstriction, only modest increases in R5 were observed following exercise challenge [35]. At present, bronchoprovocation thresholds for clinically meaningful interpretation in both adults and children should be considered in the context of scientific research and optimal and shared thresholds must be validated in further dedicated studies. While bronchodilator testing using oscillometry holds promise, further research is necessary to fully elucidate the clinical utility of bronchoprovocation testing. The FOT has proven particularly useful in preschool-aged children, a population in which spirometry is often unreliable. Grell et al. (2023) [36] found that abnormal FOT parameters, specifically R5, R5–20, R20, and Fres, measured in children under 5 years of age were predictive of spirometric impairment at school age. These findings suggest that the FOT may serve as a predictive tool for early asthma diagnosis [36]. Numerous studies have demonstrated the clinical utility of the FOT in the context of pediatric asthma. Improvements in X5 and R5–20 have been documented in children with uncontrolled asthma after one year of treatment, although these values remained inferior compared to healthy controls [37]. Similar findings were reported by Chaiwong who observed elevated R5 and R5–20 values in poorly controlled asthmatic children [38]. Furthermore, increases in R5, R5–20, X5, and Fres have been associated with poor asthma control in several studies [39,40]. Conversely, in adults, FOT findings have not consistently correlated with asthma control status [41]. The FOT not only complements spirometry but also provides a nuanced understanding of airway mechanics. Parameters such as R5 and low-frequency Xrs offer insights into both central and peripheral airway function. The FOT has shown high sensitivity in detecting bronchodilator responsiveness, though it should not be interpreted in isolation, particularly with regard to R5–20, which may not fully capture the complexity of airway obstruction. Although the FOT has not demonstrated a significant correlation with fractional exhaled nitric oxide (FeNO), it has shown considerable promise in the detection of small airway dysfunction (SAD). SAD, which contributes to poor asthma control and increased exacerbation risk, often occurs even in mild asthma and may coexist with normal spirometry. Oscillometric parameters such as R5, X5, and Fres are particularly effective in identifying and quantifying SAD [42]. In a recent study, SAD was present in 73% of asthma patients and was associated with a higher rate of exacerbations, despite no differences in spirometry indices [8]. These findings align with evidence suggesting that the FOT may be superior to spirometry in diagnosing asthma and evaluating short-term treatment outcomes [30]. The FOT may play an essential role in monitoring treatment efficacy. It has been utilized to evaluate changes in airway resistance and reactance following initiation of inhaled corticosteroids (ICS) and long-acting β-agonists (LABA) [43]. Furthermore, therapies targeting small airways—such as extrafine-particle ICS/LABA combinations and biologic agents like dupilumab and benralizumab—have demonstrated measurable improvements in the FOT parameters [42]. the FOT parameters may even guide personalized therapy. Sugawara et al. demonstrated that selection of ICS based on the FOT determined airflow obstruction type (central vs. peripheral) improved therapeutic outcomes. For instance, mometasone furoate was more effective in patients with central-type obstruction, whereas fluticasone propionate yielded better results in those with peripheral-type disease, as evidenced by improvements in the Leicester Cough Questionnaire [44]. More recently, we described that in a cohort of eosinophilic severe asthma patients, FOT R5–19 values correlate with FEV1/FVC and are significantly higher in obstruction [45].

Of notice, while for individuals aged 6 years and older spirometry, whenever feasible, is still considered the primary lung function test suggested by international guidelines, Global Initiative for Asthma (GINA) recognizes that spirometry may be challenging in younger children who cannot perform forced expiratory maneuvers. GINA acknowledges that alternative methods—including the FOT/oscillometry—are increasingly available and can be considered, especially in children of preschool age who are unable to reliably perform spirometry [46,47].

### 3.2. Chronic Obstructive Pulmonary Disease

Chronic Obstructive Pulmonary Disease (COPD) is a chronic respiratory disorder characterized by persistent respiratory symptoms and airflow limitation due to airway and/or alveolar abnormalities, typically caused by significant exposure to noxious particles or gases. According to the Global Initiative for Chronic Obstructive Lung Disease (GOLD), the hallmark features of COPD include chronic cough, sputum production, dyspnea, and recurrent respiratory infections, often associated with bronchitis, bronchiolitis, and emphysematous destruction [48]. The diagnosis of COPD is primarily based on spirometry, which demonstrates a post-bronchodilator ratio of forced expiratory volume in one second (FEV_1_) to forced vital capacity (FVC) of less than 0.70 or below the lower limit of normal, indicating persistent airflow limitation [49]. The FOT has emerged as a valuable non-invasive tool to complement spirometry in the assessment of COPD. As known, the FOT is particularly useful for detecting early changes in peripheral airways, which are often involved before spirometry abnormalities become evident. Parameters such as R5, the difference between resistance at 5 and 20 Hz (R5–20), and the area under the reactance curve (AX) have shown strong correlations with disease severity, symptom burden, and exacerbated risk in COPD patients [50]. For example, Baba et al. (2022) demonstrated that patients with smoking-related COPD exhibit greater impairment in oscillometric indices of small airways and a reduced bronchodilator response compared to non-smokers [51]. Furthermore, the FOT has shown promising results in identifying subclinical airway impairment in asymptomatic smokers with normal spirometry, supporting its potential role in screening high-risk populations for early COPD detection [38]. The frequency-dependent nature of oscillometry measurements allows for the detection of subtle changes in small airways, which are often the earliest site of pathology in COPD. This makes the FOT particularly valuable in the early stages of disease, when traditional spirometry may still appear normal. Several studies have also highlighted the ability of the FOT to differentiate between COPD and other obstructive lung diseases such as asthma. Notably, Desai and Joshi reported a significant association between increased ΔX and COPD severity, suggesting its utility in distinguishing COPD from asthma [52]. Brashier and Salvi further observed that in asthma, resistance (R) is more prominently affected, while in COPD, reactance (X) and parameters reflecting peripheral airway involvement (e.g., R5–20 and AX) are more consistently impaired. This was corroborated by studies showing that even at similar levels of FEV_1_, COPD patients tend to show greater abnormalities in peripheral airway indices compared to asthmatic patients [53]. Incorporating the FOT into the routine assessment of COPD patients might thus offer a more comprehensive evaluation of disease burden.

## 4. Application of the Forced Oscillatory Technique in Restrictive Lung Diseases

### 4.1. Interstitial Lung Diseases

Interstitial Lung Diseases (ILDs) encompass a heterogeneous group of pulmonary disorders characterized by varying degrees of inflammation and fibrosis of the lung interstitium [54]. Spirometric evaluation typically reveals a restrictive ventilatory defect, with reductions in forced vital capacity (FVC), total lung capacity (TLC), and diffusing capacity for carbon monoxide (DLCO), reflecting impaired lung compliance and parenchymal distortion.

The FOT has emerged as a promising, non-invasive modality for assessing respiratory mechanics in ILDs. Although the FOT has been primarily adopted in the evaluation of obstructive airway diseases, its applicability has expanded to restrictive pathologies, offering insight into SAD, lung stiffness, and ventilation heterogeneity.

Recent histopathological and imaging studies have demonstrated that early small airway involvement—such as loss of terminal bronchioles and fibrotic remodeling—is a fundamental feature of idiopathic pulmonary fibrosis (IPF), the prototypical form of ILD [55]. This has stimulated interest in the FOT as a tool for early detection and phenotyping of ILD. Several studies have investigated correlations between FOT-derived parameters, conventional pulmonary function tests (PFTs), and high-resolution computed tomography (HRCT) findings. The FOT’s key advantage lies in its effort-independent nature, making it especially useful in elderly or debilitated patients who may be unable to perform reliable spirometry [56]. A retrospective analysis by Takeichi et al. involving 29 patients with various respiratory conditions indicated that oscillometric indices of impedance primarily reflect small airway alterations and ventilation inhomogeneities rather than gross parenchymal destruction. Notably, increased resistance (Rrs) and more negative reactance (Xrs) values were significantly associated with SAD in ILD patients [57]. Complementary findings by Mikamo et al. revealed significant alterations in R5–20, X5, resonant frequency (Fres), and area of reactance (AX) in ILD patients with SAD [58]. In a comparative study, Miyoshi et al. evaluated FOT parameters across ILD, asthma, and COPD cohorts. ILD patients exhibited lower resistance values (R5, R20) than those with asthma yet showed more negative reactance and higher Fres and ALX values than COPD patients. These findings underscore the potential of the FOT in distinguishing restrictive from obstructive phenotypes [59]. Further, Xrs has emerged as a sensitive marker of restrictive impairment. Tatsuru Ishikawa et al. identified a significant association between baseline Xrs and survival in patients with IPF, with specific cut-off values proposed to predict three-year mortality. Their study highlights the prognostic potential of the FOT in longitudinal disease monitoring [9]. Changes in inspiratory X5 have been associated with FVC and DLCO, as shown by Matesanz-López et al., reinforcing the role of the FOT as a surrogate marker of disease severity. Notably, unlike in COPD, where expiratory reactance dominates oscillometric profiles due to dynamic airway collapse, ILD is characterized by more pronounced abnormalities during inspiration, an important distinction with potential diagnostic implications [56]. The FOT has also shown utility in the assessment of connective tissue disease-associated ILD (CTD-ILD). In rheumatoid arthritis-related ILD, a resonant frequency above 14.14 Hz was significantly associated with greater disease burden on HRCT and served as an independent predictor of pulmonary involvement [60]. Additionally, Panagopoulos et al. demonstrated that in systemic sclerosis-associated ILD (SSc-ILD), SAD could be detected via the FOT even in the absence of HRCT abnormalities, with the R5–20 gradient serving as a sensitive marker of subclinical airway involvement [61,62].

Finally, application of the FOT might be relevant to assess lung function in rare and ultra-rare diseases with lung involvement, such as Acid Sphingomyelinase Disease (ASMD) which can manifest from childhood and which can be characterized by restrictive as well as mixed respiratory pattern [63,64].

### 4.2. Obesity and Kyphoscoliosis

Restrictive respiratory patterns are also observed in extrapulmonary conditions such as obesity and kyphoscoliosis, wherein thoracic mechanics are altered due to anatomical or functional constraints.

In obesity (defined as BMI > 30), increased body mass results in reduced functional residual capacity (FRC), leading to elevated airway resistance. Miura et al. demonstrated that R5 values increase proportionally with BMI, influenced both by reduced lung volumes and upper airway narrowing due to adipose tissue accumulation. The study emphasized the importance of adjusting oscillometric reference values for BMI and sex, especially when interpreting results in epidemiological or clinical settings [65]. Similarly, in kyphoscoliosis and ankylosing spondylitis, thoracic cage deformities reduce chest wall compliance and lung expansion. Using the FOT across a frequency range of 2–26 Hz, Van Noord et al. found elevated Rrs and mildly reduced Xrs in both conditions, with negative frequency dependence, reflecting mechanical restriction and inhomogeneous impedance distribution [22].

### 4.3. Neuromuscular Disorders

The FOT is emerging as a valuable, non-invasive tool for assessing respiratory mechanics in patients with neuromuscular disorders (NMDs), including amyotrophic lateral sclerosis (ALS), where traditional spirometry may be limited by poor patient cooperation or bulbar dysfunction. FOT indices, particularly low-frequency reactance (X5), showed significant correlations with conventional measures such as FVC and maximal inspiratory pressure (MIP), highlighting the FOT’s sensitivity to early respiratory muscle weakness even when spirometric values remain within normal limits. This supports its role as a screening and monitoring tool in NMDs, especially for detecting subclinical respiratory decline.

Furthermore, the FOT may help differentiate between upper and lower airway contributions to respiratory dysfunction and could be particularly beneficial in longitudinal monitoring or in individuals with bulbar involvement who are unable to perform forced maneuvers reliably.

Although standardization is still evolving, the FOT holds promise for incorporation into routine respiratory surveillance in NMD populations and may complement conventional tools in assessing the impact of interventions such as non-invasive ventilation or respiratory muscle training [66].

## 5. Future Perspectives

The FOT has transitioned from an ancillary assessment tool to a potentially key component in the diagnostic and prognostic functional evaluation of both obstructive and restrictive lung disease. Its ability to capture subtle alterations in peripheral airway function, particularly in early or subclinical disease stages, makes it highly attractive for clinical integration.

Recent studies have proposed the use of oscillometric indices such as Fres and Xrs not only for detection but also for the prognostic purposes. For instance, Fres > 14.14 Hz correlates with both HRCT-based disease extent and spirometric impairment, offering predictive accuracy superior to traditional PFTs in early rheumatoid arthritis-ILD [60]. In IPF, inspiratory Fres and ALX have been linked to disease progression and composite physiological indices [67].

Moreover, a limited number of Clinical Trials so far have focused on the FOT or impulse oscillometry outcomes as primary/secondary endpoints or biomarkers. Table 2 includes trial identifiers, target population, interventions, the FOT parameters measured, outcome role, and current status.

Standardization of the FOT thresholds, integration into multidimensional scoring systems, and validation through longitudinal cohorts will be essential for a wider adoption of this technique. Moreover, the use of machine learning algorithms could unlock the full diagnostic potential of the multifrequency, intra-breath data generated by the FOT, enabling early stratification and personalized treatment pathways.

As respiratory medicine moves toward more personalized and noninvasive diagnostic tools, the FOT might play a growing role in future guidelines for the diagnosis and management of asthma and COPD. As already evidenced, since the FOT allows for sensitive assessment of respiratory mechanics during tidal breathing without requiring patient effort or forced maneuvers, the technique might be especially beneficial in the diagnosis and follow up of elderly, pediatric, or severely ill populations. Future guidelines are expected to incorporate the FOT not only as a diagnostic adjunct but also as a tool for ongoing disease management, especially as normative reference values become standardized and device accessibility improves. Integration of the FOT into clinical pathways may enhance objective assessment in patients who are unable to perform reliable spirometry, ultimately contributing to a more inclusive and effective approach to chronic airway disease management.

## 6. Limitations of the Forced Oscillation Technique

Despite its promising advantages, the FOT still has several limitations which restrict its widespread clinical adoption. Particularly, one of the main challenges lies in the lack of universally accepted reference values and standardized protocols, which might limit the comparison of results across different devices and populations.

Furthermore, while the FOT is highly sensitive to describe peripheral airway mechanics, no standardized comparability to volumetric spirometry data limit its standalone diagnostic value in current guideline-based classifications.

Interpretation of the FOT parameters requires specialized expertise and remains less intuitive to many clinicians compared to traditional spirometry.

Large-scale, prospective trials validating the FOT impact on outcomes are needed before it can be fully integrated into routine practice or guidelines.

## 7. Economic Considerations and Global Availability of the Forced Oscillation Technique

From an economic perspective, although the initial investment in the FOT devices can be substantial, improved disease monitoring, earlier diagnosis of SAD, and consequently better disease management might reduce the costs related to disease therapy. Unfortunately, at the moment, to the best of our knowledge, no studies on the economic impact of the wide-scale adoption of the FOT are available.

The current lack of standardized reimbursement pathways and limited guideline endorsement in many health systems may hinder widespread adoption of the FOT, especially in resource-limited environments. Additionally, clinicians need to be specifically trained in the use of the FOT and the relative unfamiliarity with the FOT among general practitioners could result in FOT underutilization.

Moreover, the availability and clinical use of the FOT vary widely across countries, reflecting differences in healthcare infrastructure, guideline adoption, economic resources, and diagnostic priorities. While in high-income countries the FOT has started to be used, particularly in academic centers, as a complement to common lung function tests, both in research and selected clinical use, in low- and middle-income countries, access to the FOT is very limited. The high cost of equipment, lack of trained personnel, and focus on acute respiratory infections and tuberculosis often push chronic disease diagnostics like the FOT to a lower priority.

In this sense, overall, the global availability of the FOT is expanding, but its use remains primarily concentrated in research settings or specialized care units in wealthier countries.

## 8. Conclusions

In conclusion, the FOT offers a non-invasive, effort-independent, and highly sensitive modality for the functional characterization of obstructive and restrictive lung diseases. Although, at the moment, the FOT is mainly used for research purposes, its utility has been demonstrated in the diagnosis, monitoring, and prognostication, particularly in patients for whom conventional spirometry might be limiting or inconclusive due to the inability to coordinate or to produce an adequate effort. Future research and standardization of the technique are needed to ensure that the FOT is increasingly considered a core component in respiratory diagnostics of obstructive and restrictive lung diseases.

## Figures and Tables

**Table 1:** Schematic comparison of the main FOT parameters alterations and FOT applications in obstructive and restrictive lung diseases.

FOT Parameter	Asthma	COPD	ILD
**Z**	↑↑	↑↑	↑
**Rrs**	↑↑	↑↑↑	↑
**Xrs**	↓↓	↓↓↓	↑↓
**R5–20**	↑↑↑	↑↑↑	↑↓
**Clinical and Research Applications of the FOT**			
Complementary tool to spirometry	↑↑↑	↑↑↑	↑↑
Detection of SAD	↑↑↑	↑↑	↑↑
Bronchodilator test (research use)	↑↑	↑↑	Not needed
Bronchoprovocation test (research use)	↑↑	Not needed	Not needed
Exercise-induced bronchoconstriction test (research use)	↑↑	Not needed	Not needed

Legend: Z: impedance; Rrs: resistance; Xrs: reactance; R5–20: Difference in Resistance measured at 5 and 20 Hz. For the FOT alterations, (↑): slightly increased; (↑↑): moderately increased; (↑↑↑): highly increased; (↑↓): stable, not significantly modified; (↓↓): moderately decreased; (↓↓↓): highly decreased. For the FOT applications, (↑↑): moderately useful; (↑↑↑): highly useful. COPD: Chronic Obstructive Pulmonary Disease; ILD: Interstitial Lung Disease; R5–20: Difference in Resistance measured at 5 and 20 Hz; X5: reactance at 5 Hz; Fres: resonant frequency; AX: area under the reactance curve.

**Table 2:** Ongoing or recent trials incorporating FOT parameters.

NCT/ID	Condition/ Population	Intervention/Design	FOT Parameters (e.g., Rrs, Xrs, ΔXrs)	Role of FOT Outcome	Status/Estimated Completion
NCT07063563	COPD, pulmonary rehabilitation	Rehabilitation at moderate vs. low altitude	Rrs, Xrs, ΔXrs	Primary/Secondary	Recruiting; primary completion 31 December 2025 (ClinicalTrials.gov)
NCT01151618	COPD under mechanical ventilation	NIV with flow-limitation detection via FOT	ΔXrs (within-breath reactance at 5 Hz)	Primary	Completed (2010) (ctv.veeva.com)
NCT05612256	Mechanically ventilated newborn infants	Observational, comparing EIT + SOPI + FOT	Xrs and Rrs	Exploratory biomarkers/ composite score	Recruiting; completion estimated. December 2024 (ICHGCP)

Legend: electrical impedance tomography (EIT), saturation oxygenation pressure index (SOPI).
